# Editorial: Towards a better understanding of fruit ripening: Crosstalk of hormones in the regulation of fruit ripening

**DOI:** 10.3389/fpls.2023.1173877

**Published:** 2023-03-09

**Authors:** Xiaoyang Zhu, Qiunan Zhu, Hong Zhu

**Affiliations:** ^1^ Guangdong Provincial Key Laboratory of Postharvest Science of Fruits and Vegetables/Engineering Research Center for Postharvest Technology of Horticultural Crops in South China, Ministry of Education, College of Horticulture, South China Agricultural University, Guangzhou, Guangdong, China; ^2^ Key Laboratory of South China Agricultural Plant Molecular Analysis and Genetic Improvement & Guangdong Provincial Key Laboratory of Applied Botany, South China Botanical Garden, Chinese Academy of Sciences, Guangzhou, China

**Keywords:** phytohormones, cross-talking, postharvest, fruit ripening, regulation

The ripening and senescence of fruits are crucial to their quality and shelf life, where a number of changes occur in color, texture, flavor, aroma, nutrition, etc (Seymour et al., 2008). Hormones are important factors in the initiation and regulation of fruit ripening and senescence. Numerous studies have shown that various endogenous hormones in fruits synergically affect this process. Therefore, it is important to study the hormone regulatory mechanism that would provide insights into how to improve and maintain fruit quality and storability *via* hormone regulation.

## Ethylene, a key player in fruit ripening

The function of ethylene in regulating fruit ripening has received the most attention and been well studied (Wang et. al.), but its regulatory network in many non-model fruits is not yet fully understood. Li et al. investigated the regulation of ethylene in mango fruit ripening, using ethylene and 1-methylcyclopropene (1-MCP), the action inhibitor of ethylene.They found that MiERF2 and MiERF8 may play critical roles in regulating mango fruit softening. Wang et al. reported that blueberry had an atypical climacteric ripening process, where a respiratory climacteric character was developmentally regulated by ethylene, but with non-autocatalytic ethylene production during fruit ripening. Except for ethylene, increasing evidence show that other hormones such as auxin, abscisic acid (ABA), brassinosteroids (BRs), gibberellins (GAs), melatonin (MT) and cytokinins (CKs) control fruit ripening (Ji et al., 2021; Kou et al., 2021). Other regulatory factors, including epigenetic maintenance and modification and microRNAs, are also involved in fruit ripening regulation (Liu et al., 2015; Fenn and Giovannoni, 2021). Mo et al. showed that lysine ubiquitination plays an critical role in papaya fruit ripening. Specifically, they found the ubiquitination levels of three key proteins in ethylene signal pathway were significantly changed during the ripening period.

The variety of hormones that affect ripening and the complex interplay among them make it challenging to analyze their respective roles.In the present topic collection, we report new findings on the function of hormones in ripening regulation, focusing on the interaction between ethylene and other hormone signaling pathways.

## Interaction between ethylene and ABA

Fruits are commonly distinguished by respiration pattern and ethylene production during ripening, which can be classified as climacteric fruits such as tomato and banana, and non-climacteric fruits such as litchi and strawberry (Kou and Wu, 2018; Wang et al.). It is generally considered that ethylene is the key actor of climacteric fruit ripening and ABA is the core player of non-climacteric fruit (Kou et al., 2021; Wang et al.). However, increasing evidences have shown that fruit ripening is regulated by a balance of multiple hormones rather than by a single one, and the crosstalk between ethylene and ABA signaling plays a leading role in regulating fruit ripening. In climacteric fruits, ABA usually accumulates before the initiation of climacteric period that can promote ethylene biosynthesis *via* upregulating ethylene biosynthesis related genes and accelerating ethylene-triggered ripening (Meng et al., 2016; Álvarez-Flórez et al., 2017). Ethylene interacted with ABA to regulate fig fruit ripening (Qiao et al., 2021). In tomato, SlNAC1 regulates the expression of *SlPSY1*, *SlACS2/4* and *SlACO1*tointegrate ethylene and ABA signaling and mediate fruit ripening (Meng et al., 2016). In non-climacteric fruits like strawberry, the ripening is co-regulated by ethylene, ABA and IAA (Bai et al., 2021). The expression of *ACS/ACO*, key genes in ethylene biosynthesis, is regulated by AREB/ABF in ABA signaling, while the expression of *NCED*, is mediated by ERF. These genes may contribute to the ABA-ethylene interaction in non-climacteric fruit ripening (Bai et al., 2021).

## Crosstalk between ethylene and auxin

Auxin is an important hormone in plant development, and recent studies reveal that auxin is also involved in fruit ripening *via* interacting with other hormones, especially with ethylene (Tadiello et al., 2016). Ethylene interacted with auxin to regulate apple fruit ripening (Tadiello et al., 2016). In peach fruit, PpILR1 (IAA-leucine resistant) activated the expression of *PpACS1*and integrated auxin-ethylene signaling to regulate fruit ripening (Wang et al., 2021a). In pear fruit, PpERF4 and PpIAA1 bound to each other to form a positive feedback loop to regulate ripening process by integrating ethylene and auxin signals (Wang et al., 2021b). In papaya, auxin delayed fruit ripening *via* the interaction of CpEIL1and CpARF2, which mediated ethylene-auxin interaction to regulate fruit ripening (Zhang et al., 2020). In apple, auxin activated MdARF5 and then induced the transcription of ethylene biosynthesis related genes including *MdACS1, MdACS3a* and *MdACO1*, to initiate apple fruit ripening (Yue et al., 2020).

## JA interacts with ethylene in regulating fruit ripening

JA plays an important role in plant development, especially in plant stress response (Wasternack, 2014). Recent work also found that JA participated in fruit ripening including coloration and softening (Coelho et al., 2019). In tomato, JA treatment prevented fruit softening and maintained the accumulation of lycopene and carotenoids (Liu et al., 2018). MYC2 is an important transcriptional activator in JA signaling pathway, and MdMYC2 could bind to the promoters of *MdACO1* and *MdACS1* and enhanced their expression to promote ethylene production to regulate apple fruit ripening (Li et al., 2017). JA could also regulate ethylene biosynthesis *via* mediating the expression of genes in ethylene signaling and biosynthesis pathways by MdMYC2 (Li et al., 2017).

## Ethylene interacts with BR, SA, GA, CK and MT in regulating fruit ripening

It was found that exogenous BRs application could delay carambola fruit ripening and maintain fruit quality (Zhu et al., 2021). However, BRs treatment induced tomato fruit ripening by enhanced the ethylene production and respiration rate *via* inducing the expression of related genes (Zhu et al., 2015).It was also reported that *SlCYP90B3*,a key genes in BR biosynthesis, could control tomato fruit ripening in an ethylene-dependent pathway (Hu et al., 2020).

GA works as a negative regulator of fruit ripening, which inhibits the ethylene biosynthesis and perception during fruit ripening of tomato (Ji et al., 2021). SA regulates fruit ripening process mainly through the effect on the expression of ethylene signaling pathway (Kou et al., 2021). Melatonin regulates fruit ripening in a dose- and species-dependent manner, which mainly attributed to the interaction between melatonin and ethylene (Arnao and Hernández-Ruiz, 2020). Abbas et al. found that exogenous melatonin treatment significantly increased the floral volatile production in *H. coronarium* flowers, by regulating the expression of genes related to volatile biosynthesis pathway. Cytokinin (CK) plays a negative role in fruit ripening process (Ji et al., 2021). The application of synthetic CK on kiwifruit fruit reduced the ethylene biosynthesis and delayed fruit softening (Ainalidou et al., 2016). Liu et al. also found that CK suppressed the loss of total sugar and sucrose and the accumulation of acetic acid and ethanol in the fruit aril, reduced the malondialdehyde accumulation in the pericarp of the overripe litchi fruit. Cytokinin treatment repressed the litchi fruit ripening process and maintained fruit quality in the on-tree overripe fruit. However, more information is needed about the interaction of CKs with other hormones.

In conclusion, the exciting new findings presented in the present topic collection and other works provide a broad and in-depth understanding of hormone signal interactions in regulating fruit ripening. It is believed that increasing new knowledge will emerge in this exciting and evolving field.

## Author contributions

XZ and QZ wrote the manuscript, HZ edited and reviewed the manuscript. All authors contributed to the article and approved the submitted version.
